# *catena*-Poly[[(benzil bis{[(pyridin-2-yl)methylidene]hydrazone}-κ^4^ *N*,*N*′,*N*′′,*N*′′′)mercury(II)]-μ-chlorido-[dichloridomercury(II)]-μ-chlorido]

**DOI:** 10.1107/S1600536812025718

**Published:** 2012-06-13

**Authors:** Mehmet Akkurt, Ali Akbar Khandar, Muhammad Nawaz Tahir, Seyed Abolfazl Hosseini-Yazdi, Ghodrat Mahmoudi

**Affiliations:** a Department of Physics, Faculty of Sciences, Erciyes University, 38039 Kayseri, Turkey; b Department of Inorganic Chemistry, Faculty of Chemistry, University of Tabriz, P.O. Box 51666, Tabriz, Iran; c Department of Physics, University of Sargodha, Sargodha, Pakistan

## Abstract

In the title coordination polymer, [Hg_2_Cl_4_(C_26_H_20_N_6_)]_*n*_, one Hg^II^ ion is coordinated by four N atoms from the benzylbis((pyridin-2-yl)methyl­idenehydrazone) ligand and two Cl^−^ ions in a very distorted *cis*-HgCl_2_N_4_ octa­hedral geometry. The other Hg^II^ ion is coordinated in a distorted tetra­hedral geometry by four Cl^−^ ions. Bridging chloride ions link the Hg^II^ ions into a chain propagating in [010]: the Hg—Cl bridging bonds are significantly longer than the terminal bonds. The dihedral angle between the central benzene rings of the ligand is 83.3 (2)°. The packing is consolidated by weak C—H⋯Cl hydrogen bonds and C—H⋯π inter­actions.

## Related literature

For background to polyimine ligands, see: Bai *et al.* (2005 ▶); Chowdhury *et al.* (2003 ▶); Drew *et al.* (2006 ▶); Pal *et al.* (2000 ▶); Sun *et al.* (2006 ▶).
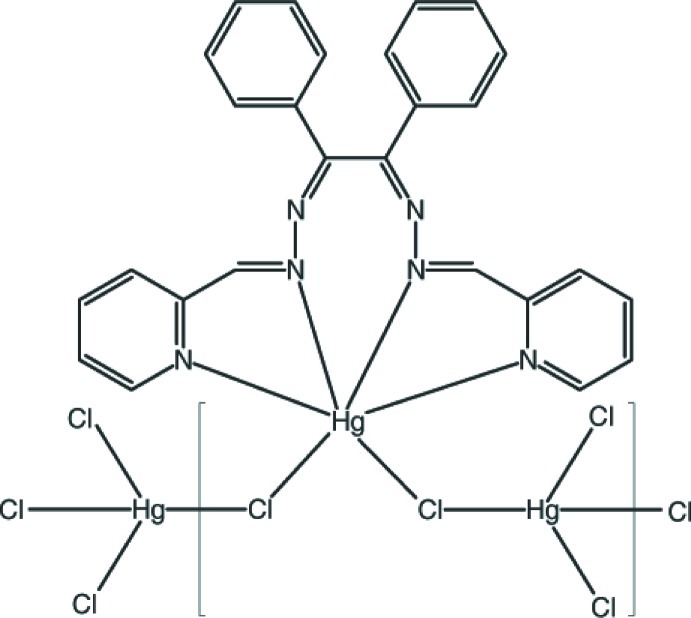


## Experimental

### 

#### Crystal data

[HgCl_4_(C_26_H_20_N_6_)]
*M* *_r_* = 959.46
Monoclinic, 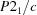

*a* = 8.8560 (2) Å
*b* = 13.8093 (4) Å
*c* = 23.6960 (7) Å
β = 96.702 (1)°
*V* = 2878.10 (14) Å^3^
*Z* = 4
Mo *K*α radiation
μ = 11.06 mm^−1^
*T* = 296 K
0.36 × 0.18 × 0.16 mm

#### Data collection

Bruker Kappa APEXII CCD diffractometer
Absorption correction: multi-scan (*SADABS*; Bruker, 2005 ▶) *T*_min_ = 0.106, *T*_max_ = 0.171
26994 measured reflections
7066 independent reflections
5295 reflections with *I* > 2σ(*I*)
*R*_int_ = 0.035

#### Refinement

*R*[*F* ^2^ > 2σ(*F* ^2^)] = 0.026
*wR*(*F* ^2^) = 0.049
*S* = 1.02
7066 reflections
343 parameters
H-atom parameters constrained
Δρ_max_ = 0.85 e Å^−3^
Δρ_min_ = −0.85 e Å^−3^

### 

Data collection: *APEX2* (Bruker, 2009 ▶); cell refinement: *SAINT* (Bruker, 2009 ▶); data reduction: *SAINT*; program(s) used to solve structure: *SHELXS97* (Sheldrick, 2008 ▶); program(s) used to refine structure: *SHELXL97* (Sheldrick, 2008 ▶); molecular graphics: *ORTEP-3 for Windows* (Farrugia, 1997 ▶) and *PLATON* (Spek, 2009 ▶); software used to prepare material for publication: *WinGX* (Farrugia, 1999 ▶) and *PLATON*.

## Supplementary Material

Crystal structure: contains datablock(s) global, I. DOI: 10.1107/S1600536812025718/hb6787sup1.cif

Structure factors: contains datablock(s) I. DOI: 10.1107/S1600536812025718/hb6787Isup2.hkl

Additional supplementary materials:  crystallographic information; 3D view; checkCIF report

## Figures and Tables

**Table 1:** Selected bond lengths (Å)

Hg1—Cl1	2.5560 (10)
Hg1—Cl2	2.5488 (11)
Hg1—N2	2.471 (3)
Hg1—N3	2.499 (3)
Hg1—N5	2.558 (3)
Hg1—N6	2.435 (3)
Hg2—Cl1	2.7777 (10)
Hg2—Cl3	2.3328 (12)
Hg2—Cl4	2.3308 (12)
Hg2—Cl2^i^	2.7227 (11)

Symmetry code: (i) 
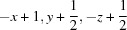
.

**Table 2:** Hydrogen-bond geometry (Å, °) *Cg*5 is the centroid of the C1–C6 phenyl ring.

*D*—H⋯*A*	*D*—H	H⋯*A*	*D*⋯*A*	*D*—H⋯*A*
C14—H14⋯Cl3^ii^	0.93	2.82	3.647 (4)	149
C25—H25⋯*Cg*5^iii^	0.93	2.96	3.814 (5)	153

Symmetry codes: (ii) 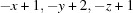; (iii) 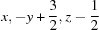.

**Fig. 1.**
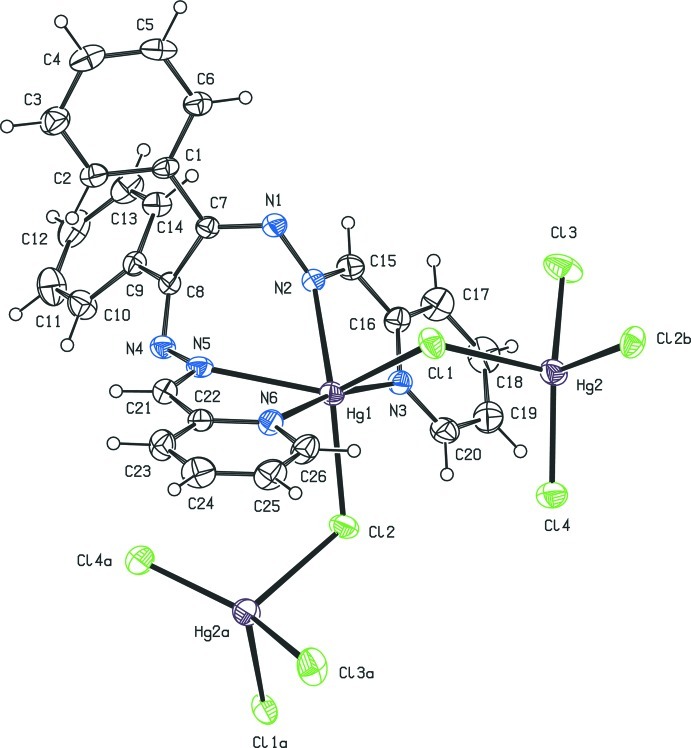
The molecular structure of the title compound with displacement ellipsoids drawn at the 30% probability level.

**Fig. 2.**
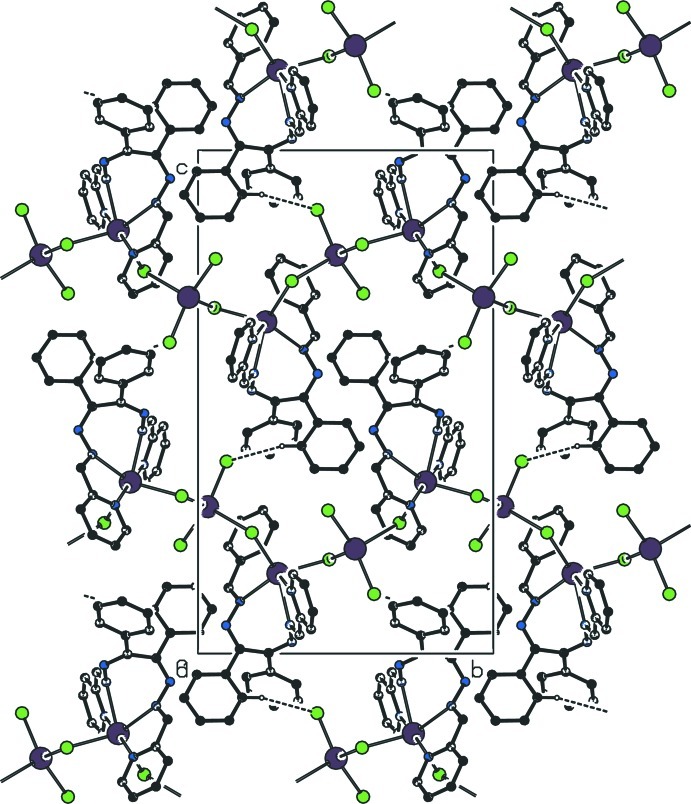
The packing and hydrogen bonding (dashed lines) of (I) viewing down a axis. H atoms not involved in hydrogen bonding are omitted.

## supplementary crystallographic information

### Comment

Research of polyimmine compound is well established currently of great interest because of their potential applications as useful organic ligands, in which the amine nitrogen atoms have strong coordination ability to transition metal ions and recongnition function (Bai *et al.*, 2005; Pal *et al.*, 2000; Chowdhury *et al.*, 2003; Drew *et al.*, 2006; Sun *et al.*, 2006). In this paper, we report the synthesis and crystal structure of the title compound.

The molecular structure of the title bimetallic coordination polymer Cl_2_Hg— Cl— (C_26_H_20_N_6_)Hg—Cl—HgCl_2_ (I) is shown in Fig. 1. Atom Hg2 is four-coordinated in a distorted tetrahedral coordination geometry by two bridging Cl atoms and two terminal Cl atoms. The bond distances of Hg—N are in the range of 2.435 (3) – 2.5558 (3) Å, the bond distances of Hg—Cl are in the range of 2.3308 (12) – 2.7777 (10) Å. *N*',*N*'-bis[1-(pyridin-2-yl)methylidene]benzil dihydrazone acts as cleating ligand here. The packing and hydrogen bonding (Table 1) is shown in Fig. 2. In addition, a C—H···π interaction contributes to the stabilization of the crystal packing.

### Experimental

Benzilbis((pyridin-2-yl)methylidenehydrazone) (*L*) was readily prepared by the reaction of benzil dihydrazone with 2-pyridinecarbpxaldehyde in a 1:2 ratio. The ligand *L* (0.5 mmol, 0.208 g) and HgCl_2_ (0.5 mmol, 0.135 g) were mixed in methanol (40 ml). The solution was left for 10 d at room temperature to afford green prisms (yield 75%). Analysis calculated for C_26_H_20_Cl_4_Hg_2_N_6_: C 66.2, H 4.0, N 11.0%; found: C 66.3, H 4.1, N 10.9%.

### Refinement

All H atoms were positioned geometrically and refined using a riding model with C—H = 0.93 Å and *U*_iso_(H) = 1.2*U*_eq_(C).

### Crystal data

**Table:**

[HgCl_4_(C_26_H_20_N_6_)]	*F*(000) = 1784
*M**_r_* = 959.46	*D*_x_ = 2.214 Mg m^−^^3^
Monoclinic, *P*2_1_/*c*	Mo *K*α radiation, λ = 0.71073 Å
Hall symbol: -P 2ybc	Cell parameters from 700 reflections
*a* = 8.8560 (2) Å	θ = 3.5–20.5°
*b* = 13.8093 (4) Å	µ = 11.06 mm^−^^1^
*c* = 23.6960 (7) Å	*T* = 296 K
β = 96.702 (1)°	Prism, green
*V* = 2878.10 (14) Å^3^	0.36 × 0.18 × 0.16 mm
*Z* = 4	

### Data collection

**Table:**

Bruker Kappa APEXII CCD diffractometer	7066 independent reflections
Radiation source: fine-focus sealed tube	5295 reflections with *I* > 2σ(*I*)
Graphite monochromator	*R*_int_ = 0.035
ω scans	θ_max_ = 28.3°, θ_min_ = 2.3°
Absorption correction: multi-scan (*SADABS*; Bruker, 2005)	*h* = −11→11
*T*_min_ = 0.106, *T*_max_ = 0.171	*k* = −18→18
26994 measured reflections	*l* = −31→31

### Refinement

**Table:**

Refinement on *F*^2^	Primary atom site location: structure-invariant direct methods
Least-squares matrix: full	Secondary atom site location: difference Fourier map
*R*[*F*^2^ > 2σ(*F*^2^)] = 0.026	Hydrogen site location: inferred from neighbouring sites
*wR*(*F*^2^) = 0.049	H-atom parameters constrained
*S* = 1.02	*w* = 1/[σ^2^(*F*_o_^2^) + (0.0162*P*)^2^ + 0.1878*P*] where *P* = (*F*_o_^2^ + 2*F*_c_^2^)/3
7066 reflections	(Δ/σ)_max_ = 0.002
343 parameters	Δρ_max_ = 0.85 e Å^−^^3^
0 restraints	Δρ_min_ = −0.85 e Å^−^^3^

### Special details

**Table:**

Geometry. Bond distances, angles *etc*. have been calculated using the rounded fractional coordinates. All su's are estimated from the variances of the (full) variance-covariance matrix. The cell e.s.d.'s are taken into account in the estimation of distances, angles and torsion angles
Refinement. Refinement on *F*^2^ for ALL reflections except those flagged by the user for potential systematic errors. Weighted *R*-factors *wR* and all goodnesses of fit *S* are based on *F*^2^, conventional *R*-factors *R* are based on *F*, with *F* set to zero for negative *F*^2^. The observed criterion of *F*^2^ > σ(*F*^2^) is used only for calculating -*R*-factor-obs *etc*. and is not relevant to the choice of reflections for refinement. *R*-factors based on *F*^2^ are statistically about twice as large as those based on *F*, and *R*-factors based on ALL data will be even larger.

### Fractional atomic coordinates and isotropic or equivalent isotropic displacement parameters (Å^2^)

**Table:**

	*x*	*y*	*z*	*U*_iso_*/*U*_eq_	
Hg1	0.62651 (2)	0.77072 (1)	0.34093 (1)	0.0365 (1)	
Hg2	0.40107 (2)	1.03230 (1)	0.29193 (1)	0.0478 (1)	
Cl1	0.68420 (11)	0.94448 (7)	0.31308 (5)	0.0508 (4)	
Cl2	0.47008 (11)	0.68234 (8)	0.25964 (5)	0.0501 (3)	
Cl3	0.37878 (15)	1.09629 (9)	0.38169 (5)	0.0692 (5)	
Cl4	0.29001 (14)	0.94051 (8)	0.21557 (5)	0.0598 (4)	
N1	0.7872 (3)	0.8179 (2)	0.47703 (13)	0.0386 (11)	
N2	0.6483 (4)	0.8111 (2)	0.44313 (12)	0.0361 (10)	
N3	0.3796 (3)	0.8157 (2)	0.37525 (13)	0.0376 (11)	
N4	0.7524 (3)	0.5949 (2)	0.44393 (12)	0.0382 (11)	
N5	0.7914 (4)	0.6422 (2)	0.39605 (12)	0.0370 (10)	
N6	0.8447 (3)	0.7191 (2)	0.29480 (13)	0.0395 (11)	
C1	0.9891 (4)	0.7451 (3)	0.53615 (14)	0.0346 (11)	
C2	1.0814 (4)	0.6645 (3)	0.54636 (16)	0.0443 (14)	
C3	1.2180 (5)	0.6703 (4)	0.58042 (18)	0.0551 (17)	
C4	1.2655 (5)	0.7574 (4)	0.60399 (18)	0.0572 (18)	
C5	1.1760 (5)	0.8389 (3)	0.59423 (16)	0.0518 (16)	
C6	1.0372 (4)	0.8329 (3)	0.56081 (15)	0.0431 (14)	
C7	0.8431 (4)	0.7388 (3)	0.49870 (14)	0.0316 (11)	
C8	0.7681 (4)	0.6415 (3)	0.49102 (15)	0.0331 (11)	
C9	0.7156 (4)	0.5941 (3)	0.54105 (15)	0.0351 (12)	
C10	0.6896 (5)	0.4958 (3)	0.54188 (18)	0.0520 (17)	
C11	0.6362 (5)	0.4532 (3)	0.5883 (2)	0.0650 (19)	
C12	0.6057 (5)	0.5077 (4)	0.6335 (2)	0.0641 (19)	
C13	0.6267 (5)	0.6055 (4)	0.63283 (17)	0.0541 (18)	
C14	0.6849 (4)	0.6493 (3)	0.58726 (16)	0.0436 (14)	
C15	0.5333 (5)	0.8437 (3)	0.46300 (16)	0.0450 (16)	
C16	0.3852 (4)	0.8468 (3)	0.42897 (15)	0.0392 (12)	
C17	0.2613 (5)	0.8829 (3)	0.45179 (19)	0.0579 (17)	
C18	0.1243 (5)	0.8889 (3)	0.4169 (2)	0.0631 (19)	
C19	0.1184 (5)	0.8602 (3)	0.3623 (2)	0.0598 (19)	
C20	0.2472 (5)	0.8220 (3)	0.34252 (18)	0.0502 (17)	
C21	0.8909 (4)	0.6034 (3)	0.36920 (16)	0.0417 (12)	
C22	0.9269 (4)	0.6436 (3)	0.31518 (15)	0.0362 (12)	
C23	1.0385 (4)	0.6025 (3)	0.28678 (18)	0.0510 (16)	
C24	1.0661 (5)	0.6424 (4)	0.23574 (19)	0.0589 (19)	
C25	0.9829 (5)	0.7200 (3)	0.21479 (18)	0.0569 (18)	
C26	0.8728 (5)	0.7570 (3)	0.24518 (17)	0.0516 (16)	
H2	1.05060	0.60550	0.52990	0.0530*	
H3	1.27800	0.61530	0.58740	0.0660*	
H4	1.35840	0.76160	0.62660	0.0690*	
H5	1.20890	0.89790	0.61010	0.0620*	
H6	0.97610	0.88760	0.55490	0.0520*	
H10	0.70800	0.45790	0.51100	0.0620*	
H11	0.62100	0.38660	0.58880	0.0780*	
H12	0.57050	0.47830	0.66480	0.0770*	
H13	0.60190	0.64300	0.66300	0.0650*	
H14	0.70330	0.71560	0.58770	0.0520*	
H15	0.54270	0.86640	0.50020	0.0540*	
H17	0.26880	0.90280	0.48950	0.0690*	
H18	0.03770	0.91240	0.43100	0.0750*	
H19	0.02840	0.86610	0.33800	0.0720*	
H20	0.24080	0.80000	0.30520	0.0600*	
H21	0.94200	0.54850	0.38400	0.0500*	
H23	1.09360	0.54930	0.30180	0.0610*	
H24	1.14100	0.61660	0.21570	0.0710*	
H25	1.00020	0.74760	0.18030	0.0690*	
H26	0.81620	0.81000	0.23070	0.0620*	

### Atomic displacement parameters (Å^2^)

**Table:**

	*U*^11^	*U*^22^	*U*^33^	*U*^12^	*U*^13^	*U*^23^
Hg1	0.0406 (1)	0.0377 (1)	0.0319 (1)	0.0028 (1)	0.0073 (1)	−0.0034 (1)
Hg2	0.0651 (1)	0.0414 (1)	0.0374 (1)	0.0024 (1)	0.0082 (1)	−0.0028 (1)
Cl1	0.0480 (6)	0.0347 (6)	0.0703 (7)	−0.0031 (5)	0.0096 (5)	0.0021 (5)
Cl2	0.0486 (6)	0.0474 (6)	0.0531 (6)	0.0031 (5)	0.0014 (5)	−0.0196 (5)
Cl3	0.1034 (10)	0.0603 (8)	0.0489 (7)	−0.0135 (7)	0.0297 (7)	−0.0188 (6)
Cl4	0.0793 (8)	0.0545 (7)	0.0430 (6)	−0.0037 (6)	−0.0032 (6)	−0.0048 (5)
N1	0.0452 (19)	0.037 (2)	0.0329 (17)	−0.0006 (16)	0.0013 (15)	−0.0003 (15)
N2	0.0432 (19)	0.0331 (18)	0.0310 (17)	0.0027 (15)	0.0001 (15)	−0.0007 (14)
N3	0.0425 (19)	0.0355 (19)	0.0342 (18)	−0.0027 (15)	0.0019 (15)	0.0019 (14)
N4	0.0486 (19)	0.0345 (19)	0.0313 (17)	−0.0030 (15)	0.0041 (15)	−0.0012 (14)
N5	0.0472 (19)	0.0383 (19)	0.0258 (16)	−0.0045 (15)	0.0051 (14)	−0.0060 (14)
N6	0.0398 (18)	0.047 (2)	0.0325 (17)	0.0084 (16)	0.0071 (14)	−0.0015 (15)
C1	0.040 (2)	0.042 (2)	0.0216 (18)	−0.0065 (18)	0.0033 (16)	0.0001 (16)
C2	0.044 (2)	0.047 (3)	0.040 (2)	−0.004 (2)	−0.0032 (19)	−0.0044 (19)
C3	0.044 (3)	0.064 (3)	0.055 (3)	0.001 (2)	−0.004 (2)	−0.002 (2)
C4	0.052 (3)	0.081 (4)	0.037 (2)	−0.015 (3)	−0.002 (2)	0.001 (2)
C5	0.065 (3)	0.057 (3)	0.034 (2)	−0.029 (2)	0.009 (2)	−0.011 (2)
C6	0.048 (2)	0.046 (3)	0.035 (2)	−0.008 (2)	0.0037 (19)	−0.0015 (19)
C7	0.0364 (19)	0.032 (2)	0.0267 (18)	−0.0028 (17)	0.0056 (16)	0.0003 (16)
C8	0.0323 (19)	0.032 (2)	0.034 (2)	0.0006 (16)	0.0002 (16)	0.0004 (17)
C9	0.034 (2)	0.036 (2)	0.035 (2)	−0.0006 (17)	0.0023 (16)	0.0039 (17)
C10	0.069 (3)	0.042 (3)	0.047 (3)	−0.005 (2)	0.015 (2)	0.003 (2)
C11	0.072 (3)	0.047 (3)	0.079 (4)	−0.005 (2)	0.021 (3)	0.022 (3)
C12	0.056 (3)	0.088 (4)	0.050 (3)	−0.001 (3)	0.013 (2)	0.031 (3)
C13	0.048 (3)	0.081 (4)	0.035 (2)	0.001 (2)	0.012 (2)	0.005 (2)
C14	0.045 (2)	0.047 (3)	0.039 (2)	−0.0078 (19)	0.0054 (19)	−0.0036 (19)
C15	0.058 (3)	0.047 (3)	0.031 (2)	0.005 (2)	0.009 (2)	−0.0040 (19)
C16	0.045 (2)	0.045 (2)	0.029 (2)	−0.0008 (19)	0.0098 (18)	0.0050 (18)
C17	0.054 (3)	0.076 (3)	0.048 (3)	0.004 (2)	0.024 (2)	0.003 (2)
C18	0.045 (3)	0.065 (3)	0.083 (4)	−0.002 (2)	0.023 (3)	0.004 (3)
C19	0.041 (3)	0.052 (3)	0.083 (4)	−0.010 (2)	−0.007 (3)	0.004 (3)
C20	0.057 (3)	0.040 (3)	0.052 (3)	−0.011 (2)	0.000 (2)	−0.001 (2)
C21	0.045 (2)	0.039 (2)	0.040 (2)	0.0019 (19)	0.0000 (19)	0.0002 (19)
C22	0.035 (2)	0.038 (2)	0.035 (2)	−0.0014 (17)	0.0018 (17)	−0.0053 (17)
C23	0.042 (2)	0.058 (3)	0.054 (3)	0.016 (2)	0.010 (2)	−0.001 (2)
C24	0.048 (3)	0.078 (4)	0.054 (3)	0.005 (2)	0.020 (2)	−0.012 (3)
C25	0.061 (3)	0.077 (4)	0.035 (2)	0.005 (3)	0.015 (2)	0.001 (2)
C26	0.053 (3)	0.067 (3)	0.035 (2)	0.009 (2)	0.006 (2)	0.005 (2)

### Geometric parameters (Å, º)

**Table:**

Hg1—Cl1	2.5560 (10)	C12—C13	1.364 (8)
Hg1—Cl2	2.5488 (11)	C13—C14	1.388 (6)
Hg1—N2	2.471 (3)	C15—C16	1.458 (6)
Hg1—N3	2.499 (3)	C16—C17	1.372 (6)
Hg1—N5	2.558 (3)	C17—C18	1.389 (6)
Hg1—N6	2.435 (3)	C18—C19	1.348 (7)
Hg2—Cl1	2.7777 (10)	C19—C20	1.387 (6)
Hg2—Cl3	2.3328 (12)	C21—C22	1.464 (5)
Hg2—Cl4	2.3308 (12)	C22—C23	1.381 (5)
Hg2—Cl2^i^	2.7227 (11)	C23—C24	1.377 (6)
N1—N2	1.392 (4)	C24—C25	1.361 (7)
N1—C7	1.282 (5)	C25—C26	1.376 (6)
N2—C15	1.254 (5)	C2—H2	0.9300
N3—C16	1.339 (5)	C3—H3	0.9300
N3—C20	1.331 (5)	C4—H4	0.9300
N4—N5	1.387 (4)	C5—H5	0.9300
N4—C8	1.282 (5)	C6—H6	0.9300
N5—C21	1.265 (5)	C10—H10	0.9300
N6—C22	1.330 (5)	C11—H11	0.9300
N6—C26	1.337 (5)	C12—H12	0.9300
C1—C2	1.385 (6)	C13—H13	0.9300
C1—C6	1.391 (6)	C14—H14	0.9300
C1—C7	1.483 (5)	C15—H15	0.9300
C2—C3	1.376 (6)	C17—H17	0.9300
C3—C4	1.371 (7)	C18—H18	0.9300
C4—C5	1.380 (7)	C19—H19	0.9300
C5—C6	1.385 (6)	C20—H20	0.9300
C7—C8	1.501 (6)	C21—H21	0.9300
C8—C9	1.476 (5)	C23—H23	0.9300
C9—C10	1.377 (6)	C24—H24	0.9300
C9—C14	1.387 (5)	C25—H25	0.9300
C10—C11	1.379 (6)	C26—H26	0.9300
C11—C12	1.362 (7)		
			
Cl1—Hg1—Cl2	111.21 (4)	C9—C14—C13	120.0 (4)
Cl1—Hg1—N2	92.64 (7)	N2—C15—C16	121.4 (3)
Cl1—Hg1—N3	93.41 (7)	N3—C16—C15	116.6 (3)
Cl1—Hg1—N5	131.59 (8)	N3—C16—C17	123.3 (3)
Cl1—Hg1—N6	88.05 (7)	C15—C16—C17	120.1 (4)
Cl2—Hg1—N2	145.31 (8)	C16—C17—C18	118.1 (4)
Cl2—Hg1—N3	86.90 (7)	C17—C18—C19	119.2 (4)
Cl2—Hg1—N5	106.34 (7)	C18—C19—C20	119.7 (4)
Cl2—Hg1—N6	84.86 (7)	N3—C20—C19	122.1 (4)
N2—Hg1—N3	66.18 (11)	N5—C21—C22	121.0 (4)
N2—Hg1—N5	71.43 (9)	N6—C22—C21	116.7 (3)
N2—Hg1—N6	122.06 (11)	N6—C22—C23	122.5 (3)
N3—Hg1—N5	118.39 (10)	C21—C22—C23	120.8 (4)
N3—Hg1—N6	171.59 (10)	C22—C23—C24	118.3 (4)
N5—Hg1—N6	65.83 (10)	C23—C24—C25	119.4 (4)
Cl1—Hg2—Cl3	99.87 (4)	C24—C25—C26	119.2 (4)
Cl1—Hg2—Cl4	101.12 (4)	N6—C26—C25	122.1 (4)
Cl1—Hg2—Cl2^i^	89.80 (3)	C1—C2—H2	119.00
Cl3—Hg2—Cl4	147.23 (5)	C3—C2—H2	119.00
Cl2^i^—Hg2—Cl3	101.86 (4)	C2—C3—H3	120.00
Cl2^i^—Hg2—Cl4	103.09 (4)	C4—C3—H3	120.00
Hg1—Cl1—Hg2	104.66 (3)	C3—C4—H4	120.00
Hg1—Cl2—Hg2^ii^	118.96 (4)	C5—C4—H4	120.00
N2—N1—C7	116.6 (3)	C4—C5—H5	120.00
Hg1—N2—N1	122.9 (2)	C6—C5—H5	120.00
Hg1—N2—C15	118.4 (3)	C1—C6—H6	120.00
N1—N2—C15	117.4 (3)	C5—C6—H6	120.00
Hg1—N3—C16	116.9 (2)	C9—C10—H10	120.00
Hg1—N3—C20	124.9 (3)	C11—C10—H10	120.00
C16—N3—C20	117.7 (3)	C10—C11—H11	120.00
N5—N4—C8	117.6 (3)	C12—C11—H11	120.00
Hg1—N5—N4	124.2 (2)	C11—C12—H12	120.00
Hg1—N5—C21	115.3 (2)	C13—C12—H12	120.00
N4—N5—C21	117.8 (3)	C12—C13—H13	120.00
Hg1—N6—C22	119.7 (2)	C14—C13—H13	120.00
Hg1—N6—C26	121.3 (2)	C9—C14—H14	120.00
C22—N6—C26	118.4 (3)	C13—C14—H14	120.00
C2—C1—C6	118.8 (3)	N2—C15—H15	119.00
C2—C1—C7	120.9 (4)	C16—C15—H15	119.00
C6—C1—C7	120.4 (4)	C16—C17—H17	121.00
C1—C2—C3	121.1 (4)	C18—C17—H17	121.00
C2—C3—C4	119.8 (5)	C17—C18—H18	120.00
C3—C4—C5	120.3 (4)	C19—C18—H18	120.00
C4—C5—C6	120.1 (4)	C18—C19—H19	120.00
C1—C6—C5	120.0 (4)	C20—C19—H19	120.00
N1—C7—C1	117.3 (3)	N3—C20—H20	119.00
N1—C7—C8	124.7 (3)	C19—C20—H20	119.00
C1—C7—C8	117.9 (3)	N5—C21—H21	120.00
N4—C8—C7	123.8 (3)	C22—C21—H21	120.00
N4—C8—C9	117.7 (3)	C22—C23—H23	121.00
C7—C8—C9	118.4 (3)	C24—C23—H23	121.00
C8—C9—C10	121.2 (4)	C23—C24—H24	120.00
C8—C9—C14	120.0 (4)	C25—C24—H24	120.00
C10—C9—C14	118.8 (4)	C24—C25—H25	120.00
C9—C10—C11	120.4 (4)	C26—C25—H25	120.00
C10—C11—C12	120.6 (4)	N6—C26—H26	119.00
C11—C12—C13	119.9 (4)	C25—C26—H26	119.00
C12—C13—C14	120.3 (4)		
			
Cl2—Hg1—Cl1—Hg2	62.36 (4)	C16—N3—C20—C19	0.7 (6)
N2—Hg1—Cl1—Hg2	−91.91 (9)	Hg1—N3—C16—C17	173.4 (3)
N3—Hg1—Cl1—Hg2	−25.64 (8)	C20—N3—C16—C17	1.2 (6)
N5—Hg1—Cl1—Hg2	−159.25 (9)	C20—N3—C16—C15	−177.0 (4)
N6—Hg1—Cl1—Hg2	146.07 (8)	Hg1—N3—C20—C19	−170.8 (3)
N3—Hg1—N2—N1	−173.7 (3)	N5—N4—C8—C7	−7.7 (5)
N5—Hg1—N2—N1	52.1 (2)	C8—N4—N5—C21	122.7 (4)
Cl1—Hg1—N5—N4	125.2 (2)	N5—N4—C8—C9	175.4 (3)
Cl1—Hg1—Cl2—Hg2^ii^	112.83 (5)	C8—N4—N5—Hg1	−76.8 (4)
N2—Hg1—Cl2—Hg2^ii^	−116.81 (13)	Hg1—N5—C21—C22	11.5 (5)
N3—Hg1—Cl2—Hg2^ii^	−154.71 (8)	N4—N5—C21—C22	173.7 (3)
N5—Hg1—Cl2—Hg2^ii^	−36.01 (9)	Hg1—N6—C22—C21	−7.5 (4)
N6—Hg1—Cl2—Hg2^ii^	26.94 (8)	C22—N6—C26—C25	0.4 (6)
Cl2—Hg1—N5—C21	65.9 (3)	C26—N6—C22—C23	−0.7 (5)
N2—Hg1—N5—C21	−150.4 (3)	Hg1—N6—C26—C25	−171.1 (3)
N3—Hg1—N5—C21	161.3 (3)	C26—N6—C22—C21	−179.1 (3)
N6—Hg1—N5—C21	−10.5 (3)	Hg1—N6—C22—C23	170.9 (3)
Cl1—Hg1—N6—C22	147.2 (3)	C7—C1—C6—C5	−177.9 (3)
Cl1—Hg1—N2—N1	−81.1 (2)	C7—C1—C2—C3	179.1 (4)
Cl2—Hg1—N2—N1	144.20 (19)	C2—C1—C7—N1	−158.9 (3)
N5—Hg1—N3—C20	−132.0 (3)	C6—C1—C7—C8	−157.5 (3)
N5—Hg1—N6—C22	9.2 (3)	C6—C1—C7—N1	19.8 (5)
N6—Hg1—N2—N1	8.2 (3)	C6—C1—C2—C3	0.3 (6)
Cl1—Hg1—N2—C15	85.7 (3)	C2—C1—C7—C8	23.8 (5)
Cl2—Hg1—N2—C15	−49.0 (3)	C2—C1—C6—C5	0.9 (5)
N3—Hg1—N2—C15	−6.9 (3)	C1—C2—C3—C4	−1.1 (6)
N5—Hg1—N2—C15	−141.1 (3)	C2—C3—C4—C5	0.8 (6)
N6—Hg1—N2—C15	175.0 (3)	C3—C4—C5—C6	0.4 (6)
Cl1—Hg1—N3—C16	−85.6 (3)	C4—C5—C6—C1	−1.2 (6)
Cl2—Hg1—N3—C16	163.4 (3)	N1—C7—C8—C9	−113.3 (4)
N2—Hg1—N3—C16	5.8 (2)	C1—C7—C8—C9	63.8 (4)
N5—Hg1—N3—C16	56.5 (3)	C1—C7—C8—N4	−113.1 (4)
Cl1—Hg1—N3—C20	86.0 (3)	N1—C7—C8—N4	69.8 (5)
Cl2—Hg1—N3—C20	−25.1 (3)	N4—C8—C9—C14	−159.8 (3)
N2—Hg1—N3—C20	177.4 (3)	C7—C8—C9—C14	23.0 (5)
N2—Hg1—N6—C22	55.3 (3)	N4—C8—C9—C10	17.2 (5)
Cl1—Hg1—N5—C21	−73.9 (3)	C7—C8—C9—C10	−159.9 (4)
Cl1—Hg1—N6—C26	−41.4 (3)	C10—C9—C14—C13	−1.0 (6)
Cl2—Hg1—N6—C26	70.1 (3)	C8—C9—C14—C13	176.1 (4)
Cl2—Hg1—N5—N4	−95.0 (2)	C8—C9—C10—C11	−178.0 (4)
N2—Hg1—N5—N4	48.7 (2)	C14—C9—C10—C11	−1.0 (6)
N3—Hg1—N5—N4	0.4 (3)	C9—C10—C11—C12	1.2 (7)
N6—Hg1—N5—N4	−171.5 (3)	C10—C11—C12—C13	0.6 (7)
Cl2—Hg1—N6—C22	−101.3 (3)	C11—C12—C13—C14	−2.6 (7)
N2—Hg1—N6—C26	−133.3 (3)	C12—C13—C14—C9	2.9 (6)
N5—Hg1—N6—C26	−179.5 (3)	N2—C15—C16—C17	−180.0 (4)
Cl4—Hg2—Cl1—Hg1	−62.53 (5)	N2—C15—C16—N3	−1.8 (6)
Cl1^ii^—Hg2^ii^—Cl2—Hg1	148.59 (5)	N3—C16—C17—C18	−1.2 (6)
Cl3^ii^—Hg2^ii^—Cl2—Hg1	−111.36 (5)	C15—C16—C17—C18	176.8 (4)
Cl4^ii^—Hg2^ii^—Cl2—Hg1	47.22 (6)	C16—C17—C18—C19	−0.6 (6)
Cl3—Hg2—Cl1—Hg1	92.17 (5)	C17—C18—C19—C20	2.4 (6)
Cl2^i^—Hg2—Cl1—Hg1	−165.83 (4)	C18—C19—C20—N3	−2.5 (6)
C7—N1—N2—Hg1	−85.4 (3)	N5—C21—C22—C23	178.3 (4)
N2—N1—C7—C1	−178.5 (3)	N5—C21—C22—N6	−3.3 (5)
C7—N1—N2—C15	107.7 (4)	C21—C22—C23—C24	179.1 (4)
N2—N1—C7—C8	−1.4 (5)	N6—C22—C23—C24	0.8 (6)
N1—N2—C15—C16	175.1 (3)	C22—C23—C24—C25	−0.4 (6)
Hg1—N2—C15—C16	7.5 (5)	C23—C24—C25—C26	0.0 (7)
Hg1—N3—C16—C15	−4.7 (4)	C24—C25—C26—N6	0.0 (7)

Symmetry codes: (i) −*x*+1, *y*+1/2, −*z*+1/2; (ii) −*x*+1, *y*−1/2, −*z*+1/2.

### Hydrogen-bond geometry (Å, º)

Cg5 is the centroid of the C1–C6 phenyl ring.

**Table:**

*D*—H···*A*	*D*—H	H···*A*	*D*···*A*	*D*—H···*A*
C14—H14···Cl3^iii^	0.93	2.82	3.647 (4)	149
C25—H25···*Cg*5^iv^	0.93	2.96	3.814 (5)	153

Symmetry codes: (iii) −*x*+1, −*y*+2, −*z*+1; (iv) *x*, −*y*+3/2, *z*−1/2.
